# The Impact of Social Media Engagement on Adult Self-Esteem: Implications for Managing Digital Well-Being

**DOI:** 10.3390/healthcare14030326

**Published:** 2026-01-28

**Authors:** Ismini Chrysoula Latsi, Alexandra Anna Gasparinatou, Nikolaos Kontodimopoulos

**Affiliations:** 1 Department of Informatics and Telematics, Harokopio University, 177 78 Athens, Greece; ap22011@hua.gr (I.C.L.); alegas@hua.gr (A.A.G.); 2 Department of Economics and Sustainable Development, Harokopio University, 176 76 Athens, Greece

**Keywords:** social media use, self-esteem, digital well-being, engagement patterns, adult populations, survey research

## Abstract

**Background/Objectives:** Social media’s impact on adult well-being varies by engagement patterns, highlighting the need for evidence to inform digital well-being strategies. This study examines the association between social media use and self-esteem, a key psychological indicator linked to adult well-being, with the aim of identifying modifiable behavioral targets relevant to clinical, workplace, and public health contexts. **Methods:** A cross-sectional survey of 81 Greek adults assessed daily social media use, engagement patterns, and self-esteem using the Rosenberg Self-Esteem Scale. Analyses included linear and exploratory quadratic regression models, multiple regression with demographic covariates (age, gender), and descriptive group comparisons. **Results:** A small but statistically significant negative association was observed between daily social media use and self-esteem (R^2^ = 0.078), indicating limited explanatory power. Exploratory analyses did not provide strong evidence of non-linear effects. Demographic factors and usage categories were not significant predictors, likely reflecting limited statistical power. Participant self-reports highlighted potentially disruptive patterns such as intensive use at specific times/conditions, perceived sleep impact, and cognitive preoccupation with social media, as well as motivation to reduce or stop use. **Conclusions:** Time spent online is a weak predictor of self-esteem, underscoring the importance of engagement quality over frequency. From a management perspective, the findings support shifting attention from generic screen-time reduction to targeting specific potentially high-risk patterns of engagement in future policy and practice. This exploratory pilot study provides initial, hypothesis-generating evidence within a Greek adult sample and highlights the need for larger, population-based studies to confirm and extend these findings.

## 1. Introduction

### 1.1. Background and Purpose of the Research

Social media platforms have become an integral part of everyday life, reshaping how individuals communicate, access information, and construct social identities. Commonly defined as Web 2.0–based platforms that enable the creation and exchange of user-generated content, social media facilitate the formation of networked communities and interactive online environments [1,2,3,4,5]. Through features such as self-presentation, connectivity, and constant availability, these platforms intersect closely with key dimensions of mental health and well-being.

Mental health is widely understood as a multidimensional construct encompassing emotional, cognitive, and social components, including affective states, life satisfaction, self-esteem, and the quality of interpersonal relationships [6,7,8,9,10]. Social media use may influence these dimensions in both positive and negative ways. On the one hand, online interaction can support social connection, information seeking, and a sense of belonging. On the other hand, patterns such as passive consumption, social comparison, cognitive preoccupation, and sleep-disruptive engagement may contribute to psychological strain, sleep disturbance, and reduced well-being.

Within this broader context, growing evidence suggests that the effects of social media are not uniform but depend on how platforms are used, the purposes they serve, and user characteristics such as social support, individual vulnerabilities, and broader social determinants [9,10,11,12,13]. While the present study does not assess all dimensions of mental health, it focuses on self-esteem as a theoretically grounded and empirically established psychological outcome through which these broader processes may be reflected. Consequently, understanding social media’s role in adult well-being requires attention not only to time spent online but also to patterns of engagement and subjective experience.

The study is guided by an integrative theoretical perspective that conceptualizes social media as interactive environments in which identity expression, social comparison, and relationship maintenance are continuously negotiated [3,4,5]. Within this framework, self-esteem is treated as a psychological outcome shaped by users’ engagement patterns, rather than as a fixed vulnerability trait or mediating mechanism. Consistent with prior literature distinguishing active, socially meaningful use from passive or comparison-based consumption, the study focuses on how specific engagement behaviors relate to variations in adult self-esteem, aligning the theoretical framework with the empirical design.

From a theoretical standpoint, the relationship between social media use and self-esteem can be understood through models emphasizing social comparison, identity construction, and feedback processes within networked environments. Social media platforms provide ongoing opportunities for self-evaluation relative to others, potentially influencing self-esteem depending on the nature and quality of engagement. While reciprocal or bidirectional pathways are theoretically plausible, the present study adopts a framework in which engagement patterns are examined as correlates of self-evaluative outcomes rather than causal determinants. This framework guides interpretation of the findings and situates them within broader debates on digital interaction and psychological well-being.

In this study, self-esteem is examined as a central psychological indicator of mental health, given its close links to social comparison, identity formation, and subjective well-being in adult populations. Against this theoretical background, the purpose of the present study is to examine the relationship between social media use and adult self-esteem, using self-esteem as a theoretically grounded outcome through which broader well-being processes may be reflected. Using a quantitative survey design, the study analyzes continuous measures of time spent on social media alongside demographic characteristics and self-reported user experiences. By focusing on young adults and working individuals—a group comparatively underrepresented in studies integrating social media use with self-esteem outcomes relative to the extensive literature on adolescents and narrowly defined adult outcomes [11,14,15,16,17,18]—this research aims to identify both potential risks and positive aspects of social media use. Accordingly, the study does not aim to assess adult mental health comprehensively, but focuses on self-esteem as a specific, empirically established psychological outcome.

From a health service and organizational management perspective, these distinctions are particularly important. Identifying specific engagement patterns that can be modified, rather than focusing solely on time spent online, supports the design of more targeted and actionable interventions. The study therefore aims to generate evidence that can inform digital well-being strategies, workplace mental health policies, and public health guidelines aimed at mitigating risks and harnessing the positive potential of social media use among adult populations.

Accordingly, the objectives of the present study are: (a) to examine the association between daily social media use and adult self-esteem; (b) to assess how specific self-reported engagement experiences (e.g., intensive use at specific times/conditions, cognitive preoccupation, and perceived sleep impact) relate to self-esteem; and (c) to explore whether sociodemographic characteristics, such as age, gender, and employment status, are associated with variations in self-esteem or condition these relationships. These objectives are addressed using regression-based analyses and group comparisons within an exploratory design.

Based on the theoretical framework and prior empirical evidence, the following hypotheses were formulated:

**H1:**  *Higher levels of daily social media use are associated with lower levels of adult self-esteem.*

**H2:**  *Self-reported engagement experiences (e.g., intensive use at specific times/conditions, cognitive preoccupation, and perceived sleep impact) are negatively associated with self-esteem.*

**H3:**  *Sociodemographic characteristics (age, gender, employment status) are associated with variation in self-esteem, and may condition the association between social media use and self-esteem.*

These hypotheses are examined within an exploratory framework and are not intended to imply causal relationships.

### 1.2. Literature Review

Existing literature provides substantial evidence on the association between social media use and adult mental health, while also revealing considerable variability in findings. Beyond individual empirical studies, several conceptual frameworks support interpretation of these relationships. Social media platforms are characterized by varying degrees of self-presentation and social presence, as well as functional components such as identity, conversation, sharing, presence, relationships, reputation, and groups [1,2]. These features reflect the broader transition to Web 2.0 environments, where users simultaneously produce and consume content and develop a “networked self” [3,4]. Social network sites, in particular, facilitate the creation of public or semi-public profiles and networks of connections that may enhance social capital [5,19].

Mental health is commonly conceptualized as a multidimensional construct encompassing emotional, cognitive, and social well-being, all of which may be influenced positively or negatively by online interaction [6,7,8,10]. Reviews and meta-analyses consistently indicate that the impact of social media use on mental health is not inherently positive or negative but depends on patterns and contexts of use.

Koh et al. [14] reviewed over 60 studies published since 2015 and reported mixed effects of social media use on adult mental health. Targeted use for information or communication was generally associated with improved well-being, whereas passive use was linked to negative emotional outcomes. Importantly, time spent online alone was found to be an insufficient predictor of mental health. Similarly, Ulvi et al. [11], in their analysis of studies conducted between 2010 and 2022, found that moderate use was not necessarily harmful, while heavier use was associated with depression and anxiety, particularly when demographic characteristics were considered.

Several studies have emphasized physiological and behavioral mechanisms underlying these associations. Stangl et al. [20] reported increased cortisol levels associated with social networking site use, particularly Facebook, linking notifications and constant connectivity to sleep problems, loneliness, and anxiety. Ahmed et al. [21] identified small but significant associations between social media use, depressive and anxiety symptoms, and sleep disturbances, while also highlighting the need for more longitudinal evidence [17,22].

Distinctions between excessive and problematic use further refine understanding of these relationships. Reviews focusing on problematic use show consistent negative associations with psychological and subjective well-being, whereas high usage alone produces less consistent effects [23,24,25,26,27]. During the COVID-19 period, Wong et al. [17] found that overall associations between social media use and well-being weakened over time, underscoring the influence of broader contextual stressors.

### 1.3. Empirical Evidence

Empirical studies provide further insight into how motives, engagement quality, and user characteristics shape mental health outcomes. Cross-sectional research from Thailand demonstrated that targeted use for social connection and information was associated with higher psychological well-being, while self-promotion and prolonged passive use increased negative emotions [12]. Longitudinal evidence suggests that high-quality interaction may produce small but positive effects on subjective and psychological well-being over time [28].

Other studies have examined underlying mechanisms and moderating factors. Research using structural equation modeling highlighted the dual role of social media in fostering social capital while also contributing to feelings of isolation [13]. Studies focusing on vulnerable populations indicate that individuals with pre-existing depression or anxiety experience disproportionately negative effects even at comparable levels of use [15]. Usage-pattern analyses further show that problematic or highly connected patterns are associated with increased depressive and anxiety symptoms [16].

Cyberbullying has emerged as a key mediating mechanism linking social media use with adverse mental health outcomes, with variations across age and gender groups [29]. Similarly, research on social media addiction consistently links compulsive use with depression, stress, and anxiety, particularly among working adults [24]. Longitudinal population-based studies reinforce the importance of engagement type, showing that active use is associated with more favorable mental health outcomes than passive use, especially among individuals with limited offline support [30]. Daily diary studies further suggest that high-quality social support can buffer negative emotional effects associated with increased use [31].

### 1.4. Critical Evaluation of the Literature and Research Gap

Across reviews and empirical studies, a consistent conclusion is that the manner of social media use is more important for mental health than frequency alone. Targeted, interactive use is generally associated with positive outcomes, whereas passive and comparison-based use is linked to negative emotions and reduced well-being [11,14,19,28,30,31,32]. Longitudinal evidence further emphasizes that the quality of online relationships outweighs time spent online in predicting well-being [28,30,31], echoing broader findings on the role of supportive relationships in mental health [6,7,8,9,10].

Problematic use, characterized by compulsivity and emotional dependence, is more consistently associated with adverse outcomes than excessive use per se [18,21,23,24,25,26]. Mechanisms such as sleep disturbance, cyberbullying, and social comparison play a central role in explaining these effects [15,16,20,21,29]. At the same time, demographic and sociocultural factors shape vulnerability and coping resources, as evidenced by studies conducted in diverse cultural contexts [9,11,12,13,15,29].

Despite extensive research, important gaps remain. Many studies focus primarily on time spent online without integrating behavioral mechanisms, platform characteristics, or contextual factors. Evidence is also heavily based on Western samples, limiting generalizability. The present study addresses these gaps by providing a micro-level analysis of adult social media use in Greece, combining descriptive data with regression-based approaches to examine both positive and negative dimensions of use. Rather than asking whether social media affects adult well-being broadly, this study focuses on which aspects of use are associated with self-esteem as a specific psychological outcome, highlighting the role of individual, cultural, and contextual differences [9,10,11,12,13].

Beyond confirming established associations, this study makes three specific contributions to the literature. First, it provides empirical evidence from a Greek adult sample, addressing the limited representation of Southern European contexts in research largely based on North American and Northern European populations. Second, it focuses on young and working adults, a group that has received comparatively less attention in studies integrating social media use with self-esteem outcomes, relative to the extensive literature on adolescents. Although adult social media use has been widely examined—particularly in relation to workplace distraction, professional networking, and platform-specific behaviors—fewer studies integrate engagement quality, subjective user experience, and self-esteem within non-clinical adult samples. The present study addresses this gap by examining how specific patterns of social media engagement relate to self-esteem in everyday, non-clinical contexts.

Third, the study combines continuous measures of time spent on social media with self-reported engagement patterns and subjective experiences, allowing for a more nuanced assessment of how social media use is linked to self-esteem. By situating these analyses within a distinct sociocultural context, the study extends existing findings and enhances their cross-cultural relevance rather than merely replicating prior work [9,11,14]. Accordingly, the study does not aim to capture all dimensions of adult mental health, but focuses on self-esteem as a theoretically grounded and empirically robust outcome linked to social media engagement patterns.

Focusing on the Greek adult population allows examination of social media–self-esteem associations within a sociocultural context that is underrepresented in the literature. Given differences in social norms, family structures, and patterns of digital engagement across countries, extending analysis to Greece strengthens the external validity of existing findings and helps determine whether associations observed in predominantly Northern European and North American samples generalize to other cultural settings.

## 2. Materials and Methods

### 2.1. Research Design

The study employed a quantitative, cross-sectional survey design to investigate the relationship between social media use and adult self-esteem.

### 2.2. Sample and Data Collection

The sample comprised 81 adult social media users residing in Greece, aged 18–57 years, who completed an electronic questionnaire. The survey was distributed via institutional email accounts at Harokopio University, personal email contacts, and student networks using Google Forms. Participants were recruited through convenience sampling, primarily within an educational context. As such, the sample should not be considered representative of the general adult population, and the findings should be interpreted as exploratory and context-specific rather than broadly generalizable. Recruitment through academic networks resulted in a higher proportion of educated individuals; although some participants were employed, the sample should not be considered representative of working adults or of the Greek population as a whole. Broader social determinants of mental health, such as socioeconomic status, were not directly measured but may have influenced the outcomes [9,10].

Although the questionnaire was intentionally brief to facilitate participation, response rates remained modest. The survey remained open for several weeks and was disseminated through multiple channels; however, participation was voluntary and uncompensated, which likely contributed to the limited final sample size. Consequently, the achieved sample reflects practical recruitment constraints rather than selective exclusion.

Although recruitment occurred primarily through academic networks for accessibility reasons, the study does not conceptualize academics or students as a distinct analytical group. Social media use and its potential implications for self-esteem are relevant across the adult population, and the present sample reflects a pragmatic entry point rather than a theoretically defined population of interest.

### 2.3. Instruments

#### 2.3.1. Social Media Use Questionnaire

The Social Media Use Questionnaire was self-developed for this study and comprised six single-item self-report questions assessing (a) frequency of social media use (from “I don’t use” to “Daily”), (b) average daily time spent on social media (hours per day), (c) cognitive preoccupation with social media (from “Never” to “Always”), (d) whether participants experience specific times or conditions in which their use becomes more intense (Yes/No), (e) perceived impact of social media use on sleep (from “Never” to “Very much”), and (f) whether participants have considered limiting or stopping social media use for mental health reasons. Most items used categorical or Likert-type response options, with one continuous estimate for daily hours. These items were intended to capture subjective perceptions and experiences rather than to serve as comprehensive behavioral measures. Given the exploratory nature of the study, no formal reliability or validity testing was conducted for this questionnaire.

For transparency, the item assessing whether participants use social media more intensively at specific hours or under specific conditions was measured using a dichotomous response format (Yes/No). Perceived sleep impact was assessed with a five-point ordinal response scale (Never, A little, Moderately, A lot, Very much). Cognitive preoccupation (“I think about social media all the time even when I don’t use it”) was assessed using a four-level frequency scale (Never, Rarely, Sometimes, Often). Intentions to limit or stop use for mental health reasons were assessed using three response options (No, Yes—considered, Yes—already done so).

#### 2.3.2. Rosenberg Self Esteem Scale (RSES)

For the respondent profile section, the Rosenberg Self-Esteem Scale (RSES) was used. The RSES is a widely applied psychological instrument designed to assess global self-esteem [33]. It consists of 10 items reflecting overall feelings of self-worth and self-acceptance, rated on a four-point Likert scale ranging from 0 (“strongly disagree”) to 3 (“strongly agree”), with higher scores indicating higher self-esteem. Positively worded items are scored directly, while negatively worded items are reverse-coded to produce a total self-esteem score. The scale assesses individuals’ general evaluation of their worth, and its psychometric properties, including reliability and validity, have been supported across multiple studies, including item response theory analyses [34].

The RSES was selected because it is one of the most widely used and extensively validated measures of global self-esteem in adult populations, allowing comparability with a large body of existing research. Its brevity and strong psychometric properties make it well suited to survey-based research with practical constraints. While alternative self-esteem or broader mental health scales exist, the use of a single, well-established instrument was considered appropriate for the exploratory aims of the present study.

In the present study, the RSES demonstrated good internal consistency (Cronbach’s α = 0.831), indicating satisfactory reliability in this sample. The full set of RSES items, as administered, is provided in the Supplementary Materials.

### 2.4. Statistical Analyses

Analyses included descriptive statistics (means, frequencies, and distributions), simple linear regression (with the Rosenberg Self-Esteem Scale score as the dependent variable and daily time spent on social media as the predictor), exploratory quadratic regression to assess potential non-linear associations, multiple regression models including age and gender as covariates, and ANOVA tests to examine differences in self-esteem across categorical variables (gender, employment status, and frequency-of-use categories).

All statistical analyses were conducted in Python version 3.11.2 using standard scientific libraries. Data handling and preprocessing were performed with Pandas version 2.2.3 and NumPy version 1.24.0, statistical modeling and inference were implemented using Statsmodels version 0.14.3, categorical variables were encoded using tools from scikit-learn version 1.4.2, and data visualization was conducted using Matplotlib version 3.7.5 and Seaborn version 0.11.2.

The significance threshold was set at *p* ≤ 0.05.

### 2.5. Ethical Issues

The study was approved by the Institutional Review Board (IRB) of the Department of Informatics and Telematics, Harokopio University (Approval reference number: 198/8 May 2025). The research was conducted in accordance with the ethical standards of the Declaration of Helsinki. All participants gave informed consent and were assured that participation was voluntary and could be withdrawn at any time.

## 3. Results

### 3.1. Descriptive Characteristics of Social Media Use

Participants reported regular engagement with multiple social media platforms. Most indicated daily use and maintained active profiles. In addition to time-based indicators, participants reported contextual patterns and subjective engagement experiences, including intensive use at specific times/conditions, cognitive preoccupation with social media, perceived sleep impact, and intention to reduce/stop use, based on single-item self-report measures. These descriptive findings provide context for the subsequent analyses and are consistent with engagement characteristics commonly observed among adult social media users [11,14].

### 3.2. Association Between Social Media Use and Self-Esteem

A simple linear regression analysis was conducted with self-esteem, measured by the Rosenberg Self-Esteem Scale, as the dependent variable and daily time spent on social media as the independent variable. A statistically significant negative association was observed between daily social media use and self-esteem (β < 0, R^2^ = 0.078), indicating that greater time spent online was associated with lower self-esteem. Although statistically significant, the explanatory power of the model was limited, suggesting that time spent online accounts for only a small proportion of variance in self-esteem. Detailed regression coefficients and confidence intervals are presented in Table 1.

**Figure 1:**
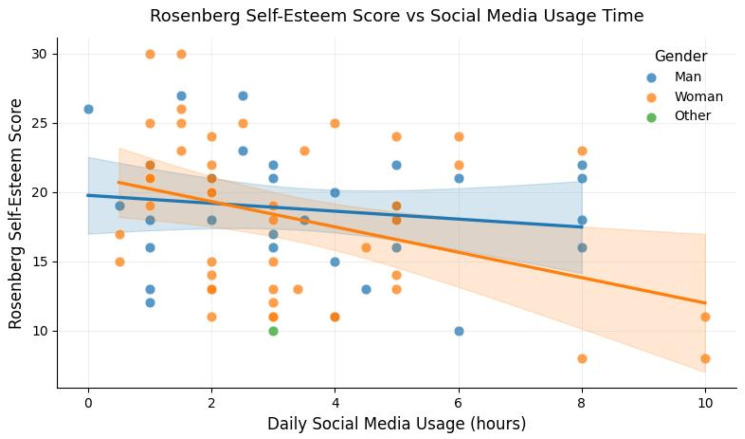
Rosenberg self-esteem score vs. daily social media use.

Figure 1 illustrates the relationship between daily time spent on social media and self-esteem scores. The regression line shows a slight downward trend, while the dispersion of data points indicates substantial variability across individuals. While the figure suggests descriptive variation in estimated self-esteem levels across gender and levels of social media use, these visual differences did not translate into statistically significant group effects or interactions in ANOVA analyses (*p* > 0.05). Given the small sample size and limited subgroup observations, the figure is intended to provide illustrative context rather than evidence of systematic or statistically reliable differences.

To explore potential non-linear associations, a second-degree (quadratic) regression model was also estimated. The quadratic term did not reach statistical significance, and the improvement in explained variance compared to the linear model was marginal. This suggests limited evidence for a non-linear relationship between time spent on social media and self-esteem in the present sample. Given the small sample size, higher-order polynomial models were not pursued to avoid overfitting. The regression estimates for the model are reported in Table 2.

### 3.3. Multiple Regression and Group Comparisons

A multiple regression model including daily time spent on social media, age, and gender was estimated to assess whether demographic factors influenced the observed association. The overall model explained 11.5% of the variance in self-esteem (R^2^ = 0.115). Time of use retained a negative association with self-esteem but was marginally non-significant (*p* = 0.071), while age and gender did not emerge as statistically significant predictors.

Group comparisons were conducted using ANOVA to examine differences in self-esteem across gender, employment status, and frequency-of-use categories. Given the small subgroup sizes, ANOVA results are interpreted descriptively. Although bootstrap resampling techniques were considered, they were not applied, as the limited sample and exploratory scope of the study would not support more robust or reliable subgroup inference. No statistically significant differences were observed in any of these comparisons (*p* > 0.05), despite some descriptive variation across groups. These findings indicate that self-esteem levels did not differ systematically across the examined demographic or usage-based categories.

### 3.4. Additional Behavioral Observations

To provide additional context for the quantitative findings, Table 3 summarizes participants’ responses to the key self-developed items assessing engagement patterns and subjective experiences.

Most participants reported more intensive use at specific times or under specific conditions (84.0%, n = 68). Regarding sleep, 37.0% (n = 30) indicated that social media affected their sleep “a lot” or “very much,” while 21.0% (n = 17) reported a moderate impact; 42.0% (n = 34) reported little or no impact. Nearly half of respondents reported having considered limiting or stopping social media use for mental health reasons (44.4%, n = 36), and 11.1% (n = 9) reported having already done so. Cognitive preoccupation with social media was reported “sometimes” or “often” by 30.9% of participants (n = 25).

## 4. Discussion

The study’s findings provide an exploratory perspective on the relationship between social media use and self-esteem in adults, rather than a comprehensive or definitive account. Linear and quadratic regression models were employed for exploratory purposes to assess whether simple associations adequately captured the relationship under study, rather than to pursue methodological complexity.

The central insight from these analyses is that time spent on social media explains only a small proportion of variance in self-esteem, reinforcing prior evidence that usage frequency alone is a weak predictor of mental health outcomes. Exploratory quadratic analyses did not provide strong evidence of non-linear effects, and any departures from linearity were modest and should be interpreted cautiously given the sample size. Overall, the findings indicate that simpler linear models capture the primary association, while exploratory extensions mainly serve to inform future research rather than add substantive explanatory power.

### 4.1. Interpretation of Findings

The study found a small but statistically significant negative association between daily social media use and self-esteem (R^2^ = 0.078). This indicates that time spent online explains only a limited portion of variance in self-esteem, consistent with international findings showing that frequency alone is a weak predictor of well-being [11,14,17,28,30,31]. This finding reinforces the view that time-based metrics capture only a narrow aspect of social media’s psychological impact. Exploratory analyses using a quadratic specification did not provide strong evidence of non-linear effects, and any deviations from linearity were modest and should be interpreted cautiously given the sample size. Overall, the results suggest that quality of engagement and individual characteristics are more informative than total time spent online, with simpler linear models capturing the main association.

Demographic variables such as age and gender were not significant predictors, aligning with mixed international evidence on demographic differences [11,12,13,14,15,29]. Rather than indicating an absence of vulnerability, these null findings suggest that demographic factors alone may be less informative than underlying psychological and behavioral processes. This pattern supports prior research indicating that individual experiences and use-related mechanisms may play a stronger role in shaping outcomes than broad demographic categories [9,15,16,18,20,21,29,32].

### 4.2. Behavioral Patterns and Mechanisms

Participants also reported contextual patterns of use and subjective experiences that complement the time-based findings. Most notably, social media use appeared to be concentrated in specific times or conditions rather than being evenly distributed across the day, consistent with evidence suggesting that the timing and context of engagement may matter more for well-being than total duration alone [20,21,25,27]. Sleep-related consequences were also commonly endorsed, consistent with prior research linking concentrated or late engagement patterns to sleep disruption, stress, and emotional fatigue [20,21,25,27]. Importantly, the present findings are descriptive and do not establish causal direction; sleep difficulties may also increase the likelihood of social media use, creating reciprocal patterns.

Participants also reported cognitive preoccupation with social media, suggesting that persistent salience of platforms may contribute to reduced self-regulation and emotional strain. Several participants also indicated motivation to reduce or stop social media use for mental health reasons, consistent with research on problematic or compulsive use and perceived loss of control [23,24,25,26]. Taken together, these observations may help explain why total daily time alone showed limited explanatory power and reinforce that the psychological impact of social media depends on how and when engagement occurs [15,16,18,27,29,32].

### 4.3. Subgroup Differences

ANOVA analyses showed no significant differences in self-esteem across gender, employment status, or frequency-of-use categories. Although international research sometimes reports gendered vulnerabilities (e.g., appearance-based comparison) [15,18,32], these patterns were not evident here. These null findings may reflect cultural context, small subgroup sizes, or unmeasured psychological differences.

The absence of effects across employment categories also suggests that work-related pressures did not strongly interact with social media use within this sample, though such interactions have been noted elsewhere [9,24]. Overall, the findings align with the broader literature emphasizing heterogeneity in user experiences.

### 4.4. Limitations

This study has several limitations that should be taken into consideration. A central limitation concerns measurement breadth, as self-esteem was the only mental health indicator assessed. While self-esteem is a well-established and theoretically relevant construct, it captures only one dimension of mental health rather than its full multidimensional scope, including anxiety, depression, or broader psychological well-being. Accordingly, the findings should be interpreted as reflecting associations between social media use and self-esteem specifically, rather than adult mental health more broadly. Incorporating indicators of anxiety, depression, or life satisfaction would provide a more comprehensive assessment of psychological well-being [6,7,8,9,10,18]. This reflects a trade-off between measurement breadth and feasibility, and future studies could benefit from employing multiple validated scales.

The sample was relatively small and convenience-based, recruited primarily within an educational setting, which introduces sampling bias and limits representativeness. The sample size should be interpreted in light of recruitment constraints and is sufficient only for exploratory analyses. Recruitment through academic networks resulted in a higher proportion of individuals with greater educational attainment, limiting the extent to which the findings can be generalized to working adults or to the Greek population as a whole. Small subgroup sizes further limited statistical power and increased the risk of Type II error; accordingly, non-significant findings in subgroup analyses (e.g., gender, employment status) should be interpreted cautiously rather than as evidence of true null effects. Given these constraints, analyses relied primarily on simpler linear models, with limited exploratory extensions interpreted cautiously. More advanced techniques, such as bootstrap resampling or structural equation modeling, were considered but deemed inappropriate due to the limited sample size and single-outcome focus. Consequently, the findings should be viewed as indicative rather than definitive and not as specific to academic populations.

All data were self-reported, which may introduce recall bias or social desirability effects, both of which are common concerns in digital behavior research [11,14,17,18,23,24]. In addition, the use of a self-developed social media use questionnaire without established psychometric properties limits measurement precision and may be affected by subjective interpretation of behaviors such as comparison or passive use.

The cross-sectional design prevents causal conclusions, as lower self-esteem may lead to increased social media use, or both may reflect underlying psychological or social factors [6,7,8,9]. Finally, the Greek sociocultural context may shape norms of self-presentation, social comparison, and technology use in ways that differ from other populations. While this limits generalizability, it also highlights the importance of culturally sensitive interpretations of social media–self-esteem relationships.

Despite these limitations, the study’s findings offer a pragmatic foundation for management considerations. The identification of potentially high-risk patterns, such as intensive use at specific times/conditions, cognitive preoccupation with social media, and self-reported sleep impact, highlights concrete targets for intervention. For health service managers, this suggests that assessing digital habits may be a useful consideration for future validated screening approaches in well-being assessments. For organizational leaders, it highlights the need to promote digital literacy and establish norms that reduce sleep-disruptive or overly salient patterns of use, thereby supporting employee well-being and productivity. Future research with larger and more diverse samples would strengthen the evidence base for such policy and practice recommendations.

### 4.5. Broader Context and Implications

Taken together, the findings are consistent with international evidence in showing that the explanatory effects of time spent on social media are limited, while more meaningful variation may be linked to patterns and contexts of use. While prior research has established that active, meaningful, and socially supportive engagement can enhance well-being, whereas passive or problematic use is more consistently associated with negative outcomes [11,14,17,18,19,20,21,23,25,28,30,31,32], the present study illustrates this distinction within a Greek adult sample using statistical associations alongside self-reported engagement experiences. Given the pilot nature of the study and the reliance on a small convenience sample and single-item self-report indicators, these implications should be interpreted as preliminary signals that require validation in larger, population-based studies before informing formal screening tools or policy implementation.

By integrating results from linear and exploratory non-linear analyses with behavioral observations, the study highlights mechanisms such as sleep disruption, cognitive preoccupation, and perceived loss of control as key explanatory pathways. These mechanisms, widely documented in the literature [15,16,20,21,23,24,25,26,27,29], help explain why time-based measures alone show weak associations with self-esteem and account for the heterogeneity observed across individuals. Sociocultural context further shapes these processes, as patterns of use and perceived norms are embedded within broader social environments [9,10,11,12,13,15,29]. Although the direction of associations aligns with international findings, examining them within a Greek context contributes to understanding how shared mechanisms operate across different cultural settings, rather than assuming uniform effects.

From a practical perspective, these findings suggest the value of moving beyond generic screen-time narratives toward more nuanced digital well-being strategies. In organizational and healthcare settings, interventions can focus on identifying high-risk patterns—such as intensive use at specific times/conditions, cognitive preoccupation with social media, and self-reported sleep impact—rather than reducing overall use indiscriminately. At the population level, public health initiatives may benefit from promoting mindful and self-regulated engagement instead of abstinence-based messaging.

Finally, emerging platform features such as algorithmic feeds and short-form video content may amplify both beneficial and harmful engagement patterns, potentially intensifying the relevance of the mechanisms identified in this study [1,2,3,27,35,36]. Overall, the findings support a balanced interpretation: social media use is neither uniformly harmful nor uniformly beneficial; its impact depends on the quality, purpose, and context of use. This integrative perspective reinforces the value of focusing on behavioral patterns and underlying mechanisms when informing research, policy, and practice.

## 5. Conclusions

This paper contributes to a clearer understanding of social media’s complex relationship with adult self-esteem and related well-being processes. The evidence indicates that while overall associations between time spent on social media and self-esteem are small, psychological outcomes vary meaningfully depending on patterns and contexts of use. Active and socially meaningful engagement may support well-being, whereas passive or problematic engagement is more frequently associated with lower self-esteem.

Key mechanisms such as disrupted sleep, social comparison, and compulsive engagement help explain these differences and highlight sources of vulnerability among adult users. Sociocultural and demographic contexts further shape user experiences, underscoring the need for context-sensitive research rather than assumptions of uniform effects.

Future research should employ longitudinal or experimental designs to better clarify causal pathways, examine mediating mechanisms in greater depth, and include larger and more diverse samples. As digital platforms continue to evolve, interdisciplinary approaches integrating insights from psychology, communication studies, and public health will be increasingly important.

Overall, a balanced perspective emerges: social media use is neither uniformly harmful nor uniformly beneficial; its impact depends on the quality, purpose, and context of engagement. This nuanced understanding provides preliminary evidence that may inform the future development and evaluation of digital well-being management strategies, including clinical guidance, workplace policies, and population-level initiatives aimed at mitigating risks and fostering beneficial use among adults.

## Figures and Tables

**Table 1:** Linear regression predicting Rosenberg self-esteem from daily time spent on social media.

	Coef.	Std. Err.	t	*p* > |t|	[0.025	0.975]
const	20.4531	1.001	20.431	0.000	18.460	22.446
Time_Usage	−0.6388	0.247	−2.587	0.012	−1.130	−0.147

**Table 2:** Detailed regression results.

Variables	Linear Regression	Quadratic Regression
Constant	20.45	20.92
x	−0.64 * (0.247)	−0.94 (0.85)
X^2^	-	0.035 (0.09)
R^2^	0.078	0.080

Notes: Values in parentheses are standard errors. * denotes significance at the 5% level.

**Table 3:** Frequencies of key self-developed items (N = 81).

Item	Response Category	n (%)
Intensive use at specific times/conditions	Yes	68 (84.0)
	No	13 (16.0)
Sleep impact attributed to social media use	Never	17 (21.0)
	A little	17 (21.0)
	Moderately	17 (21.0)
	A lot	21 (25.9)
	Very much	9 (11.1)
Considered limiting/stopping social media for mental health reasons	No	36 (44.4)
	Yes, considered	36 (44.4)
	Yes, already did so	9 (11.1)
Cognitive preoccupation with social media	Never	18 (22.2)
	Rarely	38 (46.9)
	Sometimes	16 (19.8)
	Often	9 (11.1)

## Data Availability

The raw data supporting the conclusions of this article will be made available by the authors on request.

## Supplementary Materials

The following supporting information can be downloaded at: https://www.mdpi.com/article/10.3390/healthcare14030326/s1. Supplementary file S1: Rosenberg-SES_Instrument and Instructions [33,37,38]. Supplementary file S2: Social Media Use Questionnaire.
