# Supplementary material for: The Impact of Social Media Engagement on Adult Self-Esteem: Implications for Managing Digital Well-Being
Source: Healthcare (Basel). 2026 Jan 28;14(3):326. doi: 10.3390/healthcare14030326 (PMC12897827; doi:10.3390/healthcare14030326)
Supplement: Supplementary file 1 [file healthcare-14-00326-s001.zip › Rosenberg-SES_Instrument and Instructions.pdf]

Rosenberg Self-Esteem Scale (Rosenberg, 1965)

The scale is a ten item Likert scale with items answered on a four point scale - from strongly agree to strongly disagree. The original sample for which the scale was developed consisted of 5,024 High School Juniors and Seniors from 10 randomly selected schools in New York State.

Instructions: Below is a list of statements dealing with your general feelings about yourself. If you strongly agree, circle **SA**. If you agree with the statement, circle **A**. If you disagree, circle **D**. If you strongly disagree, circle **SD**.

1.	On the whole, I am satisfied with myself.	SA	A	D	SD
2.*	At times, I think I am no good at all.	SA	A	D	SD
3.	I feel that I have a number of good qualities.	SA	A	D	SD
4.	I am able to do things as well as most other people.	SA	A	D	SD
5.*	I feel I do not have much to be proud of.	SA	A	D	SD
6.*	I certainly feel useless at times.	SA	A	D	SD
7.	I feel that I'm a person of worth, at least on an equal plane with others.	SA	A	D	SD
8.*	I wish I could have more respect for myself.	SA	A	D	SD
9.*	All in all, I am inclined to feel that I am a failure.	SA	A	D	SD
10.	I take a positive attitude toward myself.	SA	A	D	SD

Scoring: SA=3, A=2, D=1, SD=0. Items with an asterisk are reverse scored, that is, SA=0, A=1, D=2, SD=3. Sum the scores for the 10 items. The higher the score, the higher the self esteem.

The scale may be used without explicit permission. The author's family, however, would like to be kept informed of its use:

The Morris Rosenberg Foundation
c/o Department of Sociology
University of Maryland
2112 Art/Soc Building
College Park, MD 20742-1315

References

References with further characteristics of the scale:

Crandal, R. (1973). The measurement of self-esteem and related constructs, Pp. 80-82 in J.P. Robinson & P.R. Shaver (Eds), **Measures of social psychological attitudes. Revised edition**. Ann Arbor: ISR.

Rosenberg, M. (1965). **Society and the adolescent self-image**. Princeton, NJ: Princeton University Press.

Wylie, R. C. (1974). **The self-concept. Revised edition**. Lincoln, Nebraska: University of Nebraska Press.
