# Supplementary material for: The Impact of Social Media Engagement on Adult Self-Esteem: Implications for Managing Digital Well-Being
Source: Healthcare (Basel). 2026 Jan 28;14(3):326. doi: 10.3390/healthcare14030326 (PMC12897827; doi:10.3390/healthcare14030326)
Supplement: Supplementary file 1 [file healthcare-14-00326-s001.zip › Social Media Use Questionnaire.pdf]

Social Media Use Questionnaire

1. How often do you use social media (Facebook, Instagram, TikTok, etc.)
   - Daily
   - 3-6 times a week
   - 1-2 times a week
   - Less often
   - I don’t use social media

2. On average, how many hours a day do you spend on social media?

3. I think about social media all the time, even when I’m not using it.
   - Never
   - Rarely
   - Sometimes
   - Often
   - Always

4. Are there specific times or conditions when you tend to use social media more intensely?
   - Yes
   - No

5. Do you think that using social media has affected your sleep?
   - Never
   - A little
   - Moderately
   - A lot
   - Very much

6. Have you considered limiting or stopping use of social media for mental health reasons?
   - No
   - Yes, I have considered it
   - Yes, I have already done so
